# Stochastic evolutionary voluntary public goods game with punishment in a Quasi-birth-and-death process

**DOI:** 10.1038/s41598-017-16140-8

**Published:** 2017-11-23

**Authors:** Ji Quan, Wei Liu, Yuqing Chu, Xianjia Wang

**Affiliations:** 1 School of Management, Wuhan University of Technology, Wuhan, 430070 China; 2 School of Resources and Environmental Engineering, Wuhan University of Technology, Wuhan, 430070 China; 3 School of Science, Wuhan University of Technology, Wuhan, 430070 China; 4 School of Economics and Management, Wuhan University, Wuhan, 430072 China

## Abstract

Traditional replication dynamic model and the corresponding concept of evolutionary stable strategy (ESS) only takes into account whether the system can return to the equilibrium after being subjected to a small disturbance. In the real world, due to continuous noise, the ESS of the system may not be stochastically stable. In this paper, a model of voluntary public goods game with punishment is studied in a stochastic situation. Unlike the existing model, we describe the evolutionary process of strategies in the population as a generalized quasi-birth-and-death process. And we investigate the stochastic stable equilibrium (SSE) instead. By numerical experiments, we get all possible SSEs of the system for any combination of parameters, and investigate the influence of parameters on the probabilities of the system to select different equilibriums. It is found that in the stochastic situation, the introduction of the punishment and non-participation strategies can change the evolutionary dynamics of the system and equilibrium of the game. There is a large range of parameters that the system selects the cooperative states as its SSE with a high probability. This result provides us an insight and control method for the evolution of cooperation in the public goods game in stochastic situations.

## Introduction

How did human’s cooperative behavior evolve? This question is listed on the “Science” magazine as one of the 25 core problems in the 125 scientific challenges proposed by scientists all over the world^1,2^. In theory, cooperation behavior is beneficial to the group, but it is unfavorable to the individual because of the cost. Self-interested individual will choose to be a “free rider” without cooperation. The behavior of all individuals not cooperating will make the group into the most unfavorable situation. But in reality, the phenomenon of cooperation is widespread. It can be seen everywhere from human society to other biological groups in nature^3^. So, how does the cooperative behavior emerge and evolve in cooperation dilemmas? This question has been plaguing theoretical scholars from many disciplines^4^. Including the economics, sociology, psychology, theoretical biology, etc., all are trying to find some corresponding theory to explain the phenomenon of cooperation. In the study of the evolution of cooperation, the prisoner’s dilemma game and multiplayer public goods game are two basic models. In these two games, there are inconsistencies between individual’s optimal behavior and the collective optimal behavior, which depict the dilemma of cooperation in essence. At present, various cooperation mechanisms have been proposed for the cooperation dilemma in theoretical studies, such as the punishment mechanism^5–7^, the reward mechanism^8–11^, the reputation mechanism^12–15^, the network interaction mechanism^16–18^, the optional participation mechanism^19,20^, the kin selection mechanism^21,22^, the group selection mechanism^23,24^, etc. All these mechanisms can promote the evolution of cooperation under certain conditions. Nowak systematically summarized five important mechanisms which can promote cooperation^25^. With the gradual deepening on the study of these mechanisms, the evolution of cooperation will still be the focus of academic attention in the future for a long time.

This article focuses on the public goods game in both of the punishment and optional participation mechanisms which can be called voluntary public goods game with punishment. Anthropologists and biologists discovered that punishing defectors was an effective way to maintain the cooperation between human and other biological populations very long ago. By behavioral experiments, experimental economists have also found that altruistic punishment can significantly improve the level of cooperation in the population^26^. However, the reality that the punishment itself is costly for it consumes time and energy; in addition, it needs to bear the risk of other’s retaliation, which will produce the “second-order free ride” behavior. Then who will implement the punishment becomes a second-order dilemma for the problem. As Colman described that under the punishment mechanism, the interpretation of punishment is needed to replace the interpretation of cooperation^27^. Thus, various forms of peer (individual) and pool (institutional) costly punishment have been studied in the evolutionary public goods game^28–36^. For example, the introducing of punishing strategies of punishing cooperators and punishing defectors^28,29^; the introducing of the moralists who cooperate while punishing non-cooperative behavior and immoralists who defect while punishing other non-cooperative behavior^30^; tolerance-based punishment that individuals have the traits to punish the co-players based on social tolerance^33^; self-organized punishment that allowing players to adapt their sanctioning efforts in dependence on the success of cooperation^34^; conditional punishment that based the fine on the number of other punishers^35^; probabilistic sharing of the responsibility to punish defectors^36^. Chen *et al*.^37^ studied the competition and cooperation among different punishing strategies in the spatial public goods game. Chen and Perc^38^ also investigated the optimal distribution of unequal punishment for the public goods game on a scale-free network. Recent studies related to this topic include implicated punishment that punish all the group members once a wrongdoer is caught^39^; the heterogeneous punishment that divide punishers into categories according to their willing to bear for punishing^40^; competitions between prosocial punishment and exclusion^41^; etc. For a more detailed research progress on the punishment mechanism in evolutionary public goods game, the reader can refer to the recent review article of Perc *et al*.^42^.

The optional participation mechanism was first proposed by Hauert *et al*.^19^. By introducing a non-participation strategy in the game, the participant is free to choose whether or not to participate in the investment business of the public goods. The individual who does not participate does not contribute to the investment nor obtain the cooperative benefit. It is found that the introduction of non-participation strategy can resolve the cooperation dilemma in the public goods game to a certain extent. In the case of allowing non-participants, cooperation and the defection strategies can coexist. Hauert *et al*. established the replication dynamic equations of public goods game with punishment and non-participation strategies, and analyzed the evolutionary dynamics of the model^43,44^. The replication dynamic model assumes that the number of individuals in the population is infinite or large enough. It uses differential equations to describe the evolution of different strategies in the system. The evolutionary stable state of the system is analyzed by the stability of the differential equations at the equilibrium point. The stable equilibrium point is the evolutionary stable strategy (ESS) of the game. In order to describe the evolutionary dynamics in a finite size population and with mutation in the evolutionary process, Hauert *et al*. established an evolutionary public goods game model with punishment and non-participation strategies using the Moran process in the evolutionary biology^20^. Moran process based model uses the fixation probability to analyze the evolutionary dynamics of only two strategies in the system. It is generally necessary to assume weak selection in the analysis.

Because of the strong interpretability, the evolutionary voluntary public goods game based on the model of Hauert *et al*. has been widely studied by scholars. One of the extensions focuses on the use of different evolutionary dynamics to study the model. Wang and Xu *et al*. introduced the bounded rationality of the participants, using the approximate optimal reaction dynamic equations to study the evolutionary voluntary Public goods game with punishment^45,46^. Xu *et al*. introduced a self-adjustment rule for the strategy updating of individuals, and under this rule to study the evolutionary public goods game with non-participation strategy^47^, and evolutionary public goods game with both punishment and non-participation strategies^48^, respectively. Song *et al*. used the Logit evolutionary dynamics to study the cooperative behavior of groups in the public goods game with non-participation strategy^49^. It should be pointed out that all the above evolutionary dynamics studies are based on differential equations, and all the equilibrium analysis is based on the concept of the ESS.

In addition, the impacts of other factors on the cooperative behavior of the population have also been studied in the voluntary public goods game. For example, Dercole *et al*. found that under the optional participation mechanism, there was no need for excessive punishment to achieve the evolution of cooperation^50^. Rand *et al*. studied the impact of anti-social punishment on cooperative behavior of the population in the voluntary public goods game^51^. Zhong *et al*. studied the impact of the aggregation of cooperation and the liquidity of the group due to pursuit of profit on cooperative behavior of the population in the voluntary public goods game^52^. Nakamaru *et al*. studied the effects of different permit mechanisms on group cooperation and found that allowing deportation has a better effect than unconditional acceptance in promoting cooperation^53^. Valverde *et al*. studied the influence of the fluctuation on the voluntary public goods game by introducing a simple stochastic liquidity in the group interaction network^54^.

In contrast to the above studies, this paper uses the stochastic evolutionary model proposed by Amir *et al*.^55^, and studies the stochastic stable equilibrium (SSE) of voluntary public goods game with punishment in a finite size population. The concept of SSE was first proposed by Young and Foster^56,57^. Unlike ESS, SSE can effectively describe the continuous noise impact on the system during the evolutionary process of the system, which can better analyze the stability of the equilibrium in a stochastic environment. Based on the SSE, in this paper, the evolutionary process of strategies is described as a generalized quasi-birth-and-death process with ergodicity. The stochastic stable state of the system under combinations of different parameters is analyzed by the limit distribution of the stochastic process. By analyzing the SSE of the system, we can reveal the influence of these parameters on the cooperation behavior of the population in a stochastic situation.

## Results

### Voluntary Public goods game with punishment

We introduce two strategies of punishment and non-participation in the classical public goods game model. The punishment strategy participates in the investment of the public goods and punishes the defection strategies (free rides) randomly. Let *α* denote the punishment probability. Punishment is costly. Let *γ* denote the unit cost of punishment for the punisher, *β* denote the corresponding unit penalty for the individual who is punished $(\gamma  < \beta )$. Non-participation strategies do not participate in the investment nor do they get the average investment income, but can get a fixed proportion of income.

Assuming that there are four types of strategies in the population, namely cooperation type (denote as *C*), defection type (denote as *D*), punishment type (denote as *P*) and non-participation type (denote as *L*), respectively. Each time *N* individuals from the population are randomly selected to participate in the public goods game. Each individual chooses the corresponding strategy according to its type. Let *c* denote the cost of investment, and *r*the return on investment coefficient $(1 < r < N)$ of the public goods. Cooperation, defection and punishment type are collectively referred to as participation type. Let *S* (1 ≤ *S* ≤ *N*) denote the number of participation type individuals when it selected randomly from the *N* individuals. And the numbers of cooperation, defection and punishment type are *n*_*C*_, *n*_*D*_ and *n*_*P*_ respectively. When $S > 1$, the cooperation type individual’s payoff is $\frac{rc({n}_{C}+{n}_{P})}{S}-c$; the defection type individual’s payoff is $\frac{rc({n}_{c}+{n}_{p})}{S}-\alpha \beta {n}_{p}$; the punishment type individual’s payoff is $\frac{rc({n}_{C}+{n}_{P})}{S}-c-\alpha \gamma {n}_{D}$; and non-participation type individuals get a fixed income *σc* ($0 < \sigma  < r-1$). When $S=1$, there is only one participant, and the game can not happen; we assume that this participant can only be forced to obtain a fixed income as well as the non-participants.

When the population size is *M*, and the number of cooperation, defection, punishment and non-participation type individuals in the group are *i*, *j*, *k*, *l* respectively. The expected payoffs of the four types of strategies are respectively:

1 $${\pi }_{C}^{(i,j,k,l)}=\frac{(\begin{array}{c}l\\ N-1\end{array})}{(\begin{array}{c}M-1\\ N-1\end{array})}\sigma c+B-{\rm{\Phi }}(l)c\quad (i\ne 0);$$

2 $${\pi }_{D}^{(i,j,k,l)}=\frac{(\begin{array}{c}l\\ N-1\end{array})}{(\begin{array}{c}M-1\\ N-1\end{array})}\sigma c+B-\frac{k}{M-1}(N-1)\alpha \beta \quad (j\ne 0);$$

3 $${\pi }_{P}^{(i,j,k,l)}=\frac{(\begin{array}{c}l\\ N-1\end{array})}{(\begin{array}{c}M-1\\ N-1\end{array})}\sigma c+B-{\rm{\Phi }}(l)c-\frac{j}{M-1}(N-1)\alpha \gamma \quad (k\ne 0);$$

4 $${\pi }_{L}^{(i,j,k,l)}=\sigma c\quad (l\ne 0).$$

where

$$\begin{array}{c}B=\frac{rc(i+k)}{M-l-1}(1-\frac{1}{N(M-l)}(M-(l-N+1)\frac{(\begin{array}{c}l\\ N-1\end{array})}{(\begin{array}{c}M-1\\ N-1\end{array})}));\\ {\rm{\Phi }}(l)=1-\frac{r}{N}\frac{M-N}{M-l-1}+\frac{(\begin{array}{c}l\\ N-1\end{array})}{(\begin{array}{c}M-1\\ N-1\end{array})}(\frac{r}{N}\frac{l+1}{M-l-1}+r\frac{M-l-2}{M-l-1}-1).\end{array}$$

### Stochastic Evolutionary dynamics

The number of individuals in each type of strategies will evolve with the amount of their payoffs. In order to describe the evolutionary process, we introduce a stochastic process *z*(*t*). Let $z(t)=({z}_{1}(t),{z}_{2}(t),{z}_{3}(t),M-{z}_{1}(t)-{z}_{2}(t)-{z}_{3}(t))$ denote the number of cooperation, defection, punishment and non-participation type strategies in the population at time *t*, and define *z(t)* as the system state. For convenience, we abbreviate it as $({z}_{1}(t),{z}_{2}(t),{z}_{3}(t))$. The state space of the system is $S=\{(i,j,k)|0\le i+j+k\le M;i,j,k\in {\mathbb{N}}\}$, and the number of elements in the state space is $|S|=\frac{(M+1)(M+2)(M+3)}{6}$. At each time, the individual in the population adjusts its strategy according to its expected payoff, and the adjustment of the individual’s strategy leads to the change of the system state. The three assumptions: inertia, myopic and mutation in the literature^55^ about the bounded rationality of individuals in the population are used in our model. Due to inertia, we can assume that it is impossible to have more than two individuals to adjust their strategies simultaneously at one time. Myopic refers to the individual when choosing its strategy; it will only consider the current payoff, regardless of the payoff in the future. Mutation refers to the possibility that individuals may choose a non-optimal strategy with a small probability because of the complex decision-making environment and the limited nature of individual cognitive ability.

According to the above assumptions, when the system state $({z}_{1}(t),{z}_{2}(t),{z}_{3}(t))$ is $(i,j,k)\in S$, the transfer rate of the strategy *x* towards strategy *y* can be described as

5 $${p}_{x\to y}^{(i,j,k)}=\varepsilon +\kappa \cdot {({\pi }_{y}^{(i,j,k)}-{\pi }_{x}^{(i,j,k)})}^{+}\,,x,y\in \{C,D,P,L\},x\ne y.$$

where $${f}^{+}=\{\begin{array}{cc}f & f > 0\\ 0 & f\le 0\end{array},\varepsilon  > 0$$, is a small positive number, $\kappa  > 0$. For example, when ${\pi }_{x}^{(i,j,k)} > {\pi }_{y}^{(i,j,k)}$, the individual in the $(i,j,k)$ state has a more incentive to move from strategy *y* to strategy *x*, but because of the mutation, the transfer rate of the strategy *x* to strategy *y* is ${p}_{x\to y}^{(i,j,k)}=\varepsilon$. Thus, the parameter $\varepsilon$ can be seen as the noise intensity in the environment, and $\kappa$ can be understood as the speed at which the individual responds to the environment.

When *i*, *j*, *k*, *l* equals to zero respectively, the corresponding ${\pi }_{C}^{(0,j,k,l)}{\pi }_{D}^{(i,0,k,l)}{\pi }_{P}^{(i,j,0,l)}{\pi }_{L}^{(i,j,k,0)}$ does not make sense. At this point, the payoffs of cooperation, defection, punishment and non-participation type strategies are defined as the average payoff of the population.

Let $I=(i,j,k)$, $I^{\prime} =(i^{\prime} ,j^{\prime} ,k^{\prime} )$. Due to time homogeneity, let ${p}_{I,I^{\prime} }(t)$ denote the transfer probability of the system from state *I* to state $I^{\prime}$ after time *t*. That is:

6 $${p}_{I,I^{\prime} }(t)=p\{z(s+t)=(i^{\prime} ,j^{\prime} ,k^{\prime} )|z(s)=(i,j,k)\},\,\forall s > 0.$$

### Stochastic Stable Equilibrium

While $\varepsilon  > 0$, this process is ergodic. According to the properties of the stochastic process, when $t\to +\infty$, the limit of ${p}_{I,I^{\prime} }(t)$ exists and it’s independent of the initial state *I*. Let

7 $$\mathop{\mathrm{lim}}\limits_{t\to +\infty }{p}_{I,I^{\prime} }(t)={v}_{I^{\prime} }^{\varepsilon }.$$

Then ${v}_{I^{\prime} }^{\varepsilon }$ is the limit distribution of the quasi-birth-and-death process of reaching arbitrary state *I*′ $(I^{\prime} \in S)$ when the system noise is $\varepsilon$. According to the limit distribution, it is possible to determine the evolutionary stable state of the system under arbitrary noise intensity. Further, when the noise parameter is gradually reduced to zero, let

8 $$\mathop{\mathrm{lim}}\limits_{\varepsilon \to {0}^{+}}{v}_{I^{\prime} }^{\varepsilon }={v}_{I^{\prime} }.$$

According to ${v}_{I^{\prime} }$, we can determine the limit state of the system and its probability distribution when the system noise is small enough. According to Young’s description in reference^57^, state $I^{\prime} \in S$ is stochastically stable if and only if ${v}_{I^{\prime} }$ > 0.

### Numerical experiments

The Gauss-Seidel iterative algorithm introduced by Stewart in his monograph^58^ can be used to calculate the limit distribution of the above quasi-birth-and-death process. In the following, we will show the SSE of the system in our stochastic evolutionary dynamics model, and investigate the effects of game parameters on the SSE of the system. By a vast of numerical simulation experiments, we found that when $\varepsilon$ is positive and small enough, and for any parameters, the system has the limit distribution of more than zero only in the (0, 0, 0), (0, 0, 1), (0, 1, 0), (1, 0, 0), (0, *M*, 0), (*i*, 0, *M* − *i*)(*i* = 0, 1, 2, … *M*) states. According to the above definition of the SSE, only states (0, 0, 0), (0, 0, 1), (0, 1, 0), (1, 0, 0), (0, *M*, 0), (*i*, 0, *M* − *i*), (*i* = 0, 1, 2, … *M*) may be the SSEs of the system. In addition, according to the description of the model, when there is only one participation type individual in the population and whether it is cooperation or defection, this strategy is equivalent to the non-participation strategy. Thus (0, 0, 0), (0, 0, 1), (0, 1, 0), (1, 0, 0) states are essentially equivalent to the situation that all individuals choose the non-participation strategy, and we denote these states as the “All L” state. In addition, we denote the state (0, *M*, 0) as the “All D” state. States $(i,0,M-i)$ ($i=0,1,2,\ldots M$) indicate the co-existence of the cooperation and punishment strategies; or all the individuals choose the punishment strategy; or all the individuals choose the cooperation strategy, and we denote them as the “C + P” states. In the following, we fix parameters $M=20,N=4,c=1,\kappa =1,\beta =1$, and study the impacts of *α*, *γ*, *r*, *σ* on the probabilities of the system to choose different stable equilibriums.

**Figure 1:**
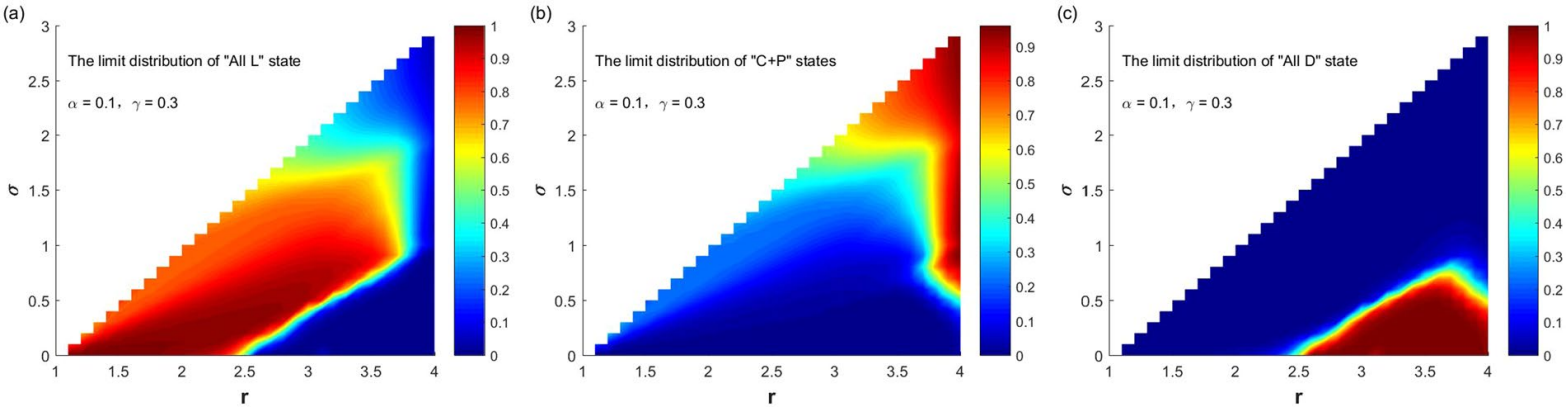
The relationship between the limit distribution of the three stable states and $(r,\sigma )$ with fixed $\alpha =0.1,\gamma =0.3$.

By numerical experiments, Fig. 1 shows the SSEs of system and the probabilities for the system to select them when punishment probability and the cost $\alpha =0.1,\gamma =0.3$, and parameters $(r,\sigma )$ continuously change in the region $1.1\le r < N,0 < \sigma  < r-1$. It can be seen from the figure, when *σ* is small and *r* is large, the system selects the “All D” state with probability 1, and only the “All D” state is the stochastic stable state of the system. When *σ* is small and *r* is small, the system selects the “All L” state with probability 1, and only “All L” state is the stochastic stable state of the system. When *σ* is slightly larger and *r* is small, the system selects the “All L” state with a large probability, and selects “C + P” states with a small probability; “All L” state and “C + P” states are the stochastic stable states of the system. In all these situations, the probability for the system choosing “C + P” states is not large, indicating that when the punishment probability *α* is small, a small fixed income coefficient *σ* has a limited effect on the cooperation behavior of the population. Only when *σ* is not too small, and *r* is large, the system selects “C + P” states with a large probability. In this situation, the effect for the promotion of cooperation is significant. Interestingly, the parameters range that the system selects different stable states with high probability is not regular. As shown in the figure, the probability that the system chooses different states exhibits rich and nonmonotonic features.

**Figure 2:**
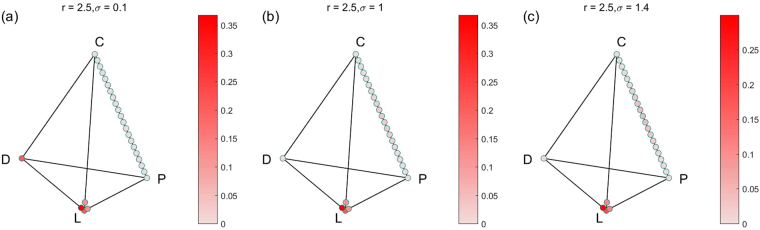
All stochastic stable states and their limit probabilities for fixed $\alpha =0.1$, $\gamma =0.3$ $r=2.5$, (**a**) $\sigma =0.1$; (**b**)$\sigma =1$; (**c**) ${\rm{\sigma }}={\rm{1.4}}$.

**Figure 3:**
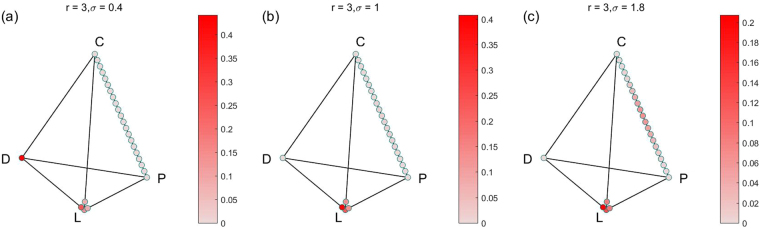
All stochastic stable states and their limit probabilities for fixed $\alpha =0.1$, $\gamma =0.3$ $r=3$, (**a**) $\sigma =0.4$; (**b**) $\sigma =1$; (**c**) ${\rm{\sigma }}={\rm{1.8}}$.

**Figure 4:**
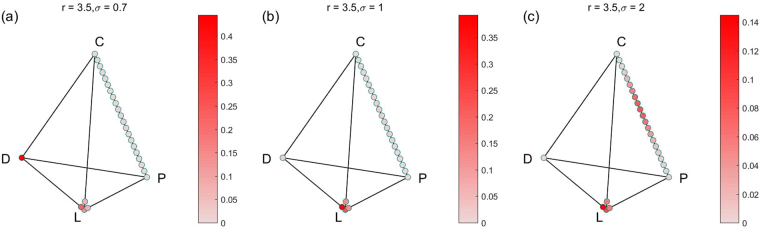
All stochastic stable states and their limit probabilities for fixed $\alpha =0.1$, $\gamma =0.3$ $r=3.5$, (**a**) $\sigma =0.7$; (**b**) $\sigma =1$; (**c**) ${\rm{\sigma }}={\rm{2}}$.

**Figure 5:**
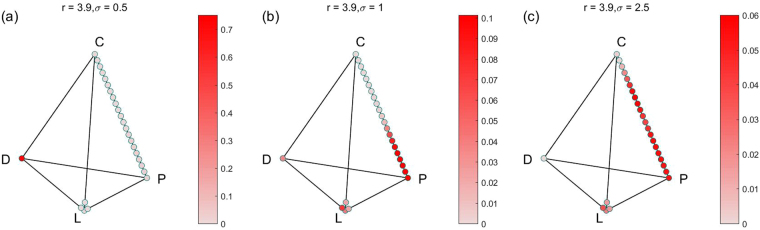
All stochastic stable states and their limit probabilities for fixed $\alpha =0.1$, $\gamma =0.3$ $r=3.9$, (**a**) $\sigma =0.5$; (**b**) $\sigma =1$; (**c**) ${\rm{\sigma }}={\rm{2.5}}$.

In order to show all the stable states and their limit distributions, we also show the results in the simplex S4 form. Because there are many parameter combinations of (*r*, *σ*), and each combination of parameters corresponds to a simplex S4. So we only choose 4 groups for 12 combinations of (*r*, *σ*) for fixed *α* = 0.1, *γ* = 0.3 to show the results. The results of the four groups are shown in Figs 2–5 respectively. For more combinations of (*r*, *σ*) as $\alpha =0.1,\,\gamma =0.3$, the possible stochastic stable states and their limit probabilities can be acquired from the supplementary attachment file named Limit Probability Data.xlsx. For every piece of data in the Excel table, we can easily draw its simplex S4.

**Figure 6:**
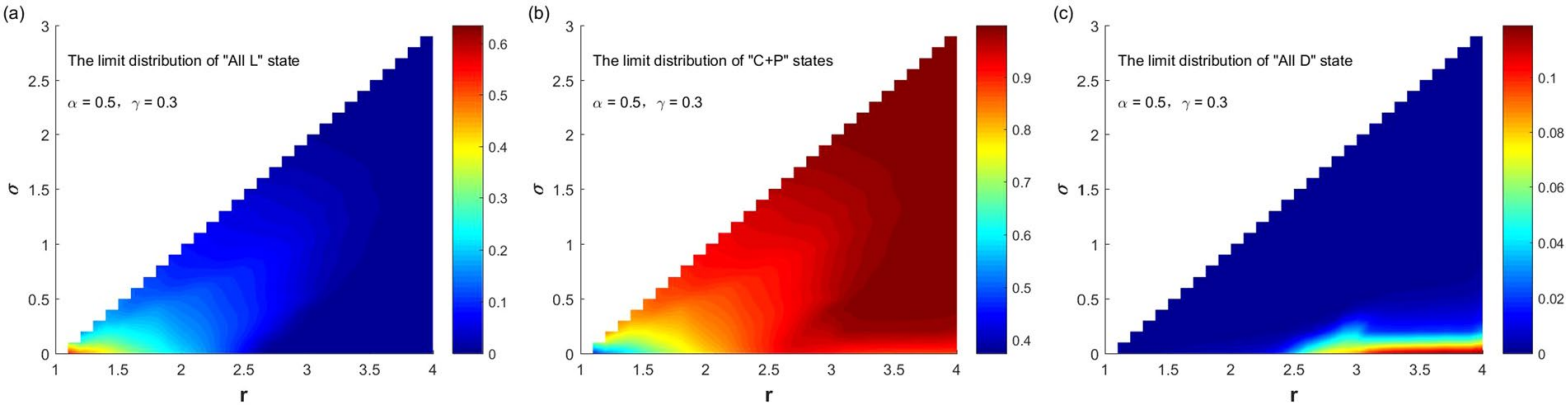
The relationship between the limit distribution of the three stable states and $(r,\sigma )$ with fixed $\alpha =0.5,\gamma =0.3$.

**Figure 7:**
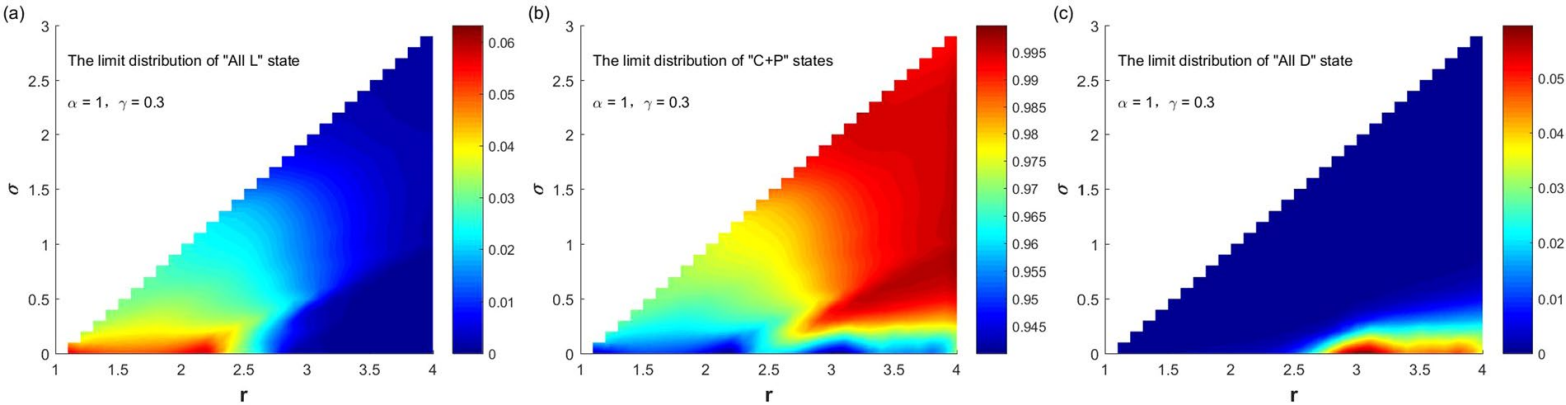
The relationship between the limit distribution of the three stable states and $(r,\sigma )$ with fixed $\alpha =1,\gamma =0.3$.

We gradually increase the parameter value of punishment probability *α*. Figure 6 shows when the punishment probability value $\alpha =0.5$ and the punishment cost $\gamma =0.3$, the SSEs of the system and the probabilities for the system to select them change with parameters $(r,\sigma )$ ($1.1\le r < N,\,0 < \sigma  < r-1$) varying. It can be seen from the figure that compared with the case $\alpha =0.1$, the increase in the punishment probability greatly expands the parameter ranges for the system selecting “C + P” states, which can greatly promote the evolution of cooperation. Except for the following two cases: for small *σ* and large *r*, the system selects the “All D” state with a small probability; for small *σ* and small *r*, the system selects the “All L” state with a not too large probability. For other parameter ranges, the system selects “C + P” states with a large probability or the probability equals to 1. If further increase the punishment probability *α*, the conclusions are similar. Figure 7 shows the corresponding case when $\alpha =1$ and $\gamma =0.3$. Compared with Fig. 6, the parameter range that the system choosing “C + P” states as its stable states has further expanded, or that the probability of choosing “C + P” states is further increased. As can be seen from the figure, for any combination of parameters, the probability of the system choosing “C + P” states is greater than 0.94, and the parameter range of the system to choose the “All D” state or the “All L” state with a small probability is consistent with Fig. 6.

**Figure 8:**
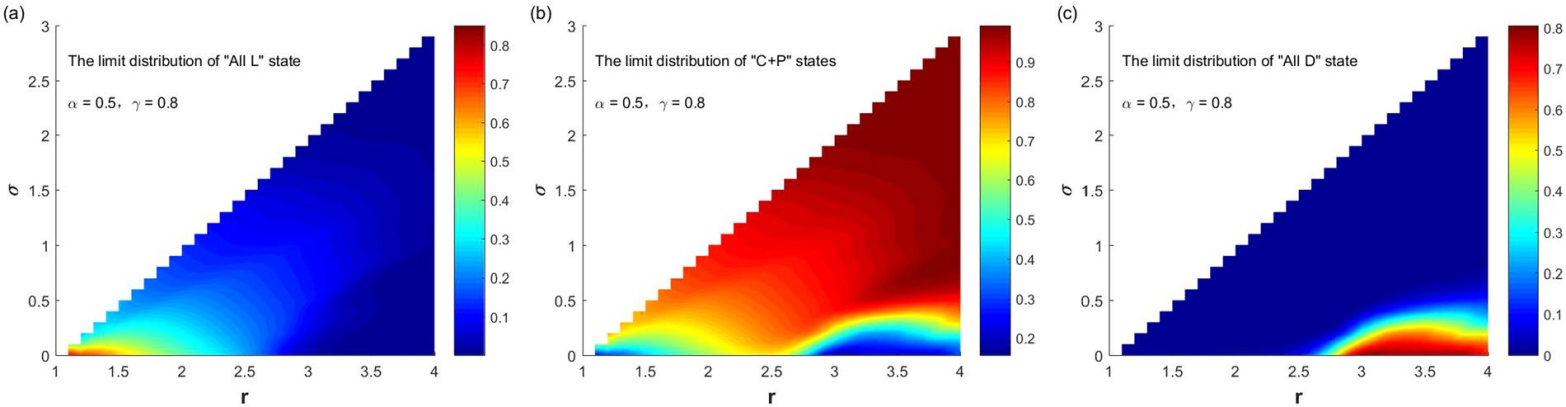
The relationship between the limit distribution of the three stable states and $(r,\sigma )$ with fixed $\alpha =0.5,\gamma =0.8$.

Finally, it is necessary to analyze the effect of the punishment cost on the system’s selection of various types of stable equilibriums and their probabilities. Take parameter values of Fig. 6 in which punishment probability and the cost $\alpha =0.5,\gamma =0.3$ as a reference. Figure 8 shows the stochastic stable equilibriums and their probabilities of the system to choose them with the change of $(r,\sigma )$ ($1.1\le r < N,0 < \sigma  < r-1$) when $\alpha =0.5$ and $\gamma =0.8$. Compared with Fig. 6, it can be found that the increase of punishment cost narrows the parameter range that system selecting the “C + P” states with high probabilities, and increases the probabilities that system selecting “All D” and “All L” states under the same parameter. For small *σ* and large *r*, the system selects the “All D” state with a large probability. For small *σ* and small *r*, the system selects the “All L” state with a large probability. It is important to note that, although the probability of punishment is not large and the cost of punishment is large, the system has a wide range of parameters of selecting “C + P” states, which also explains the effectiveness of this mechanism in promoting cooperation.

**Figure 9:**
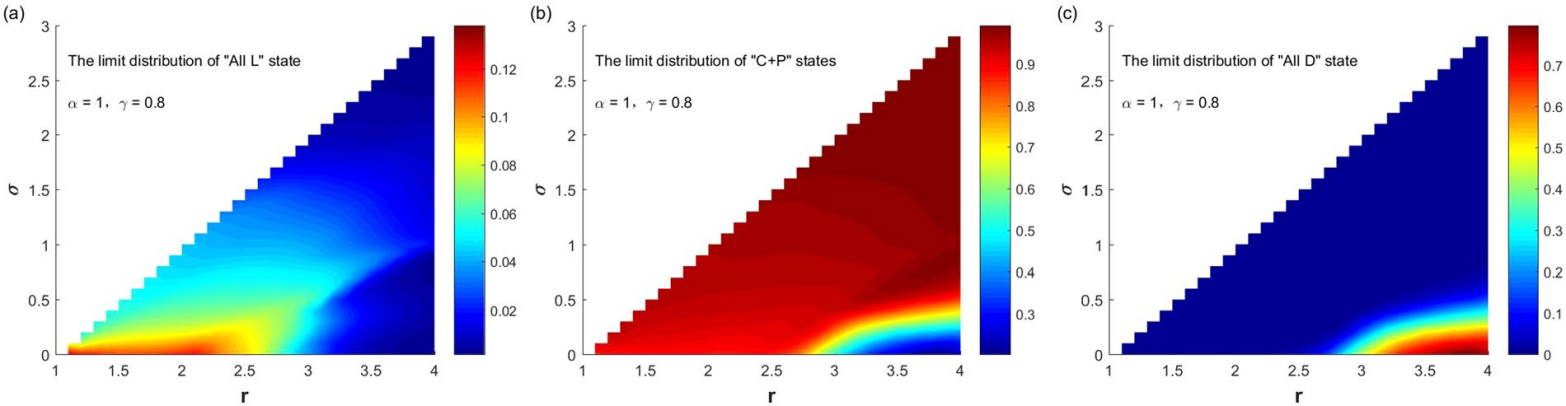
The relationship between the limit distribution of the three stable states and $(r,\sigma )$ with fixed $\alpha =1,\,\gamma =0.8$.

At the same high punishment cost, we increase the punishment probability. In order to compare the results with that in Figs 3 and 4, we let $\alpha =1,\gamma =0.8$. And Fig. 9 shows the corresponding stochastic stable equilibriums and their probabilities for the system to choose them with the change of $(r,\sigma )$ ($1.1\le r < N,0 < \sigma  < r-1$). In contrast to Fig. 7, it is found that under the same parameters, the increase in punishment costs narrows the parameter range of the system selecting “C + P” states with a large probability, and greatly increases the probability of the system selecting the “All D” state, and slightly increase the probability of the system selecting the “All L” state. When punishment cost is large, for small *σ* and large *r*, the system chooses the “All D” state with a large probability, which cannot occur for small punishment costs. In contrast to Fig. 8, it is found that when the punishment costs is high; the increase in the punishment probability is very effective for suppressing the system to choose the “All L” state; because the maximum value of ${v}_{{\rm{All}}{\rm{L}}}$ is reduced from 0.8 to near 0.14. But the suppression effect for the system to choose the “All D” state is not significant because the maximum value of the system to choose the “All D” state ${v}_{{\rm{All}}{\rm{D}}}$ is almost not changed, nor the parameter range for system to choose this state.

It can be seen from Figs 1–5 that the introduction of the punishment strategy and the non-participation strategy can change the evolutionary dynamics and equilibriums of the system in the public goods game. The system selects the “C + P” states with large probabilities over a large range of parameters. When other parameters are fixed, the influence of the four parameters that are the punishment probability *α*, the punishment cost *γ*, the fixed income coefficient of the non-participation strategy *σ*, the investment income coefficient of the public goods *r*, on the stochastic stable equilibriums of the system and their probabilities for the system to choose them can be summarized as follows: (i) For small *α* and small *γ*, there are parameter regions of $(\sigma ,r)$ for the system to choose the three stochastic stable states of the “All L”, “All D” and “C + P” states with large probabilities respectively. In general, when *σ* is small and *r* is large, the system chooses the “All D” state with a large probability; when *σ* is small and *r* is not too large, the system chooses the “All L” state with a large probability; when *σ* is not too small and *r* is large, the system chooses the “C + P” states with a large probability. The parameter regions for the system to choose these three stable states with large probabilities show some irregular features. (ii) When *γ* is small, the increase of *α* will greatly expand the parameter ranges that system selecting the “C + P” states with large probabilities, which can greatly promote the evolution of cooperation. When *α* increases to a certain extent (the specific value depends on *γ*), the system selects the “C + P” states with a probability close to 1 for any parameters of $(\sigma ,r)$. (iii) When *γ* is large, the increase of *α* can only expand the parameter range for the system choosing “C + P” states with large probabilities to a small degree. The increase in *α* is very effective for the suppression of system selecting the “All L” state, but the suppression effect of the system to choose the “All D” state is not significant. Even if *α* is increased to 1, there is still a range where the system chooses the “All D” state with a large probability. (iv) Overall, the increase of *γ* will reduce the parameter range of the system to choose “C + P” states with a large probability, and greatly increase the probability of the system choosing the “All D” state, and slightly increase the probability of the system choosing the “All L “ state under the same combination of parameters.

### Discussing

The evolution process of strategies of public goods game in a finite size population is modeled by a generalized quasi-birth-and-death process under the framework of stochastic stable equilibrium in this paper. Both punishment and non-participation strategies are introduced into the traditional public goods game. The stochastic stable state of the evolutionary system is analyzed by the limit distribution of the stochastic process. This paper focuses on the influence of parameters such as the punishment probability, the punishment cost, the fixed income coefficient of non-participation strategy, and the investment income coefficient of the public goods on the system’s selection of different equilibriums and their probabilities.

The contribution of this paper is mainly manifested in the following aspects: First, the model of this paper further develops the model of Amir *et al*.^55^, and generalizes the Markov-based evolutionary process from one-dimension to multidimensional case, while the analysis under multidimensional situation is more difficult than that in one dimension. Secondly, the model of this paper further enriches the concept of SSE. Since the concept of evolutionary games has been put forward, ESS has been regarded as its core equilibrium concept. There is little literature on the analysis of SSE in a stochastic situation. As far as we know, in the recent literature, Quan *et al*.^59^ studied the SSE of 2 × 2 symmetry stochastic evolutionary games in finite populations with non-uniform interaction rate based on a one-dimension Markov process. And other models basically based on the stochastic differential equations for analysis which assume a infinite size population, such as recent literature of Huang *et al*.^60^ and Liang *et al*.^61^. In this paper, the model is based on the multidimensional stochastic process which can describe the finite size population and the multi-strategy situation. Then, the model of this paper has different characteristics with the most commonly used Moran process model in finite size population. For the Moran process based model, it is generally necessary to assume weak selectivity in the analysis, and the fixation probability method can only analyze the evolutionary dynamic with only two kinds of strategies in the system, which has some limitations. Finally, this paper obtains the range of parameters of all possible stochastic stable equilibriums that the system may choose by numerical experiments. Among the parameters, some may not be changed according to the actual situation, but others can be adjusted within a certain range, such as the punishment probability, the fixed income coefficient. Therefore, by adjusting the corresponding parameter values, we can facilitate the system to select “C + P” states with large probabilities, which provides a feasible method for realizing the evolution and control of cooperation in public goods game.

## Methods

### Expected Payoffs in a finite size population

Assume a finite population of size *M*, the four types of strategies for cooperation, defection, punishment and non-participation are well mixed. Let $i,j,k,l$ $(i+j+k+l=M)$ denote the number of each type respectively. In the following, we analyze the expected payoff for each type of the strategies in this finite size population.

Each time *N* individuals are randomly selected from the group for the public goods game. In a sample, when the number of participation type is ${S}_{0}$, for a defection type, the probability of encountering other ${S}_{0}-1$ (${S}_{0} > 1$) participation type individuals is:

9 $$p(S={S}_{0})=\frac{(\begin{array}{c}i+j+k-1\\ {S}_{0}-1\end{array})(\begin{array}{c}l\\ N-{S}_{0}\end{array})}{(\begin{array}{c}M-1\\ N-1\end{array})}({S}_{0} > 1).$$

In these ${S}_{0}-1$ individuals, the probability that there are *m* cooperation or punishment type, and ${S}_{0}-1-m$ defection type is:

10 $$p({n}_{c}+{n}_{p}=m)=\frac{(\begin{array}{c}i+k\\ m\end{array})(\begin{array}{c}j-1\\ {S}_{0}-1-m\end{array})}{(\begin{array}{c}i+j+k-1\\ {S}_{0}-1\end{array})}.$$

We do not consider the penalty items in our analysis at first. After this treatment, the strategies of the cooperation type and the punishment type are the same, including their payoffs. When the number of participation type individuals is *S*_0_, and the total number of cooperation and punishment type individuals is *m*, then the defection type individual’s payoff is $\frac{rcm}{{S}_{0}}$ (do not consider the penalty items). Thus, for a defection type individual, when it’s in the state that the number of participation type individuals is *S*_0_ $({S}_{0} > 1)$, the expected payoff for it is:

11 $$\begin{array}{ccc}\sum _{m=0}^{{S}_{0}-1}\frac{rcm}{{S}_{0}}p({n}_{C}+{n}_{P}=m) & = & \sum _{m=0}^{{S}_{0}-1}\frac{rc}{{S}_{0}}m\frac{(\begin{array}{c}i+k\\ m\end{array})(\begin{array}{c}j-1\\ {S}_{0}-1-m\end{array})}{(\begin{array}{c}i+j+k-1\\ {S}_{0}-1\end{array})}\\  & = & \frac{rc}{{S}_{0}}({S}_{0}-1)\frac{i+k}{i+j+k-1}\end{array}$$

Therefore, when the population size is *M*, and the individual numbers of cooperation type, defection type, punishment type, non-participation type are $i,j,k,l$ respectively, the expected payoff for the defection type is (do not consider the penalty items):

12 $$\begin{array}{rcl}{\pi }_{D}^{(i,j,k,l)} & = & \sigma cp({S}_{0}=1)+\sum _{{S}_{0}=2}^{N}\frac{rc}{{S}_{0}}({S}_{0}-1)\frac{i+k}{i+j+k-1}p(S={S}_{0})\\  & = & \frac{(\begin{array}{c}l\\ N-1\end{array})}{(\begin{array}{c}M-1\\ N-1\end{array})}\sigma c+\frac{rc(i+k)}{i+j+k-1}\sum _{{S}_{0}=2}^{N}\frac{{S}_{0}-1}{{S}_{0}}\frac{(\begin{array}{c}i+j+k-1\\ {S}_{0}-1\end{array})(\begin{array}{c}l\\ N-{S}_{0}\end{array})}{(\begin{array}{c}M-1\\ N-1\end{array})}\\  & = & \frac{(\begin{array}{c}l\\ N-1\end{array})}{(\begin{array}{c}M-1\\ N-1\end{array})}\sigma c+\frac{rc(i+k)}{M-l-1}(1-\frac{1}{N(M-l)}(M-(l-N+1)\frac{(\begin{array}{c}l\\ N-1\end{array})}{(\begin{array}{c}M-1\\ N-1\end{array})}))\end{array}$$

Because when the number of participation type is ${S}_{0}$, and a participation type individual meets with other ${S}_{0}-1$ participation individuals, when its type change from the defection to the cooperation, the amount of its payoff reduction is $(1-\frac{r}{{S}_{0}})c$. Thus, in the whole population, the expected payoff difference between the defection type individuals and cooperation (or punishment) type individuals is (do not consider the penalty items):

13 $$\begin{array}{rcl}{\pi }_{D}^{(i,j,k,l)}-{\pi }_{C}^{(i,j,k,l)} & = & \sum _{{S}_{0}=2}^{N}(1-\frac{r}{{S}_{0}})c\frac{(\begin{array}{c}i+j+k-1\\ {S}_{0}-1\end{array})(\begin{array}{c}l\\ N-{S}_{0}\end{array})}{(\begin{array}{c}M-1\\ N-1\end{array})}\\  & = & (1-\frac{r}{N}\frac{M-N}{M-l-1}+\frac{(\begin{array}{c}l\\ N-1\end{array})}{(\begin{array}{c}M-1\\ N-1\end{array})}\\  &  & \times (\frac{r}{N}\frac{l+1}{M-l-1}+r\frac{M-l-2}{M-l-1}-1))c\end{array}$$

Taking into account the corresponding penalty items and penalty cost for each type of strategy and the above-mentioned expected payoffs are corrected as follows.

For defection type individuals, the penalty item brought by the punishment strategies is $\frac{k}{M-1}(N-1)\alpha \beta$. Where *α* is the probability of the defection type individuals being punished by the punishment type strategy, *β* is the penalty intensity coefficient, $\frac{k}{M-1}(N-1)$ is the expected number of punishment type individuals in a sample. For punishment type individuals, the punishment cost is $\frac{j}{M-1}(N-1)\alpha \gamma$.

### State transition probability

**Figure 10:**
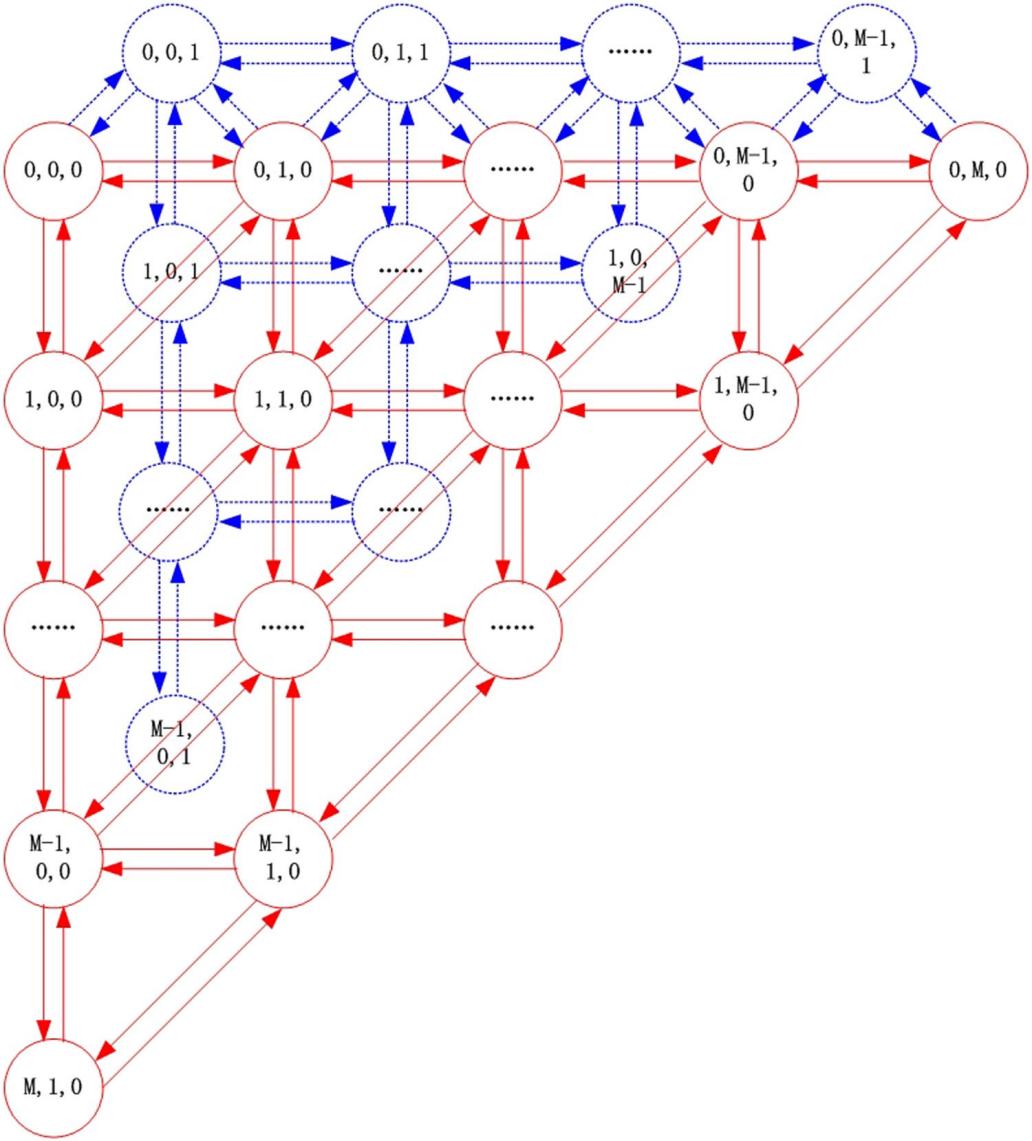
Possible state transfer processes of the system.

Figure 10 shows the possible state transfer processes of the system. Obviously, the evolution of the system is a three-dimensional state-limited and discrete Markov process; more specifically, a generalized quasi-birth-and-death process.

According to the system evolutionary rules and the transition rate between different strategies, after a small enough time *t*, the probabilities of the system transfers from state $I=(i,j,k)$ to states $(i-1,j,k+1)$, $(i-1,j,k)$, $(i-1,j+1,k)$ are ${p}_{C\to P}^{(i,j,k)}t+o(t)$, ${p}_{C\to L}^{(i,j,k)}t+o(t)$, ${p}_{C\to D}^{(i,j,k)}t+o(t)$ respectively; to states $(i,j-1,k+1)$, $(i,j-1,k)$, $(i+1,j-1,k)$ are ${p}_{D\to P}^{(i,j,k)}t+o(t)$, ${p}_{D\to L}^{(i,j,k)}t+o(t)$, ${p}_{D\to C}^{(i,j,k)}t+o(t)$ respectively; to states $(i,j+1,k-1)$, $(i,j,k-1)$, $(i+1,j,k-1)$ are ${p}_{P\to D}^{(i,j,k)}t+o(t)$, ${p}_{P\to L}^{(i,j,k)}t+o(t)$, ${p}_{P\to C}^{(i,j,k)}t+o(t)$ respectively; to states $(i+1,j,k)$, $(i,j,k+1)$, $(i,j+1,k)$ are ${p}_{L\to C}^{(i,j,k)}t+o(t)$, ${p}_{L\to P}^{(i,j,k)}t+o(t)$, ${p}_{L\to D}^{(i,j,k)}t+o(t)$ respectively; and the probability to keep the same state is $$1-{\sum }_{\begin{array}{c}x,y\in \{C,D,P,L\}\\ x\ne y\end{array}}{p}_{x\to y}^{(i,j,k)}t-o(t)$$; where *o*(*t*) is a high order infinitesimal of *t* when *t* is small enough.

**Figure:**
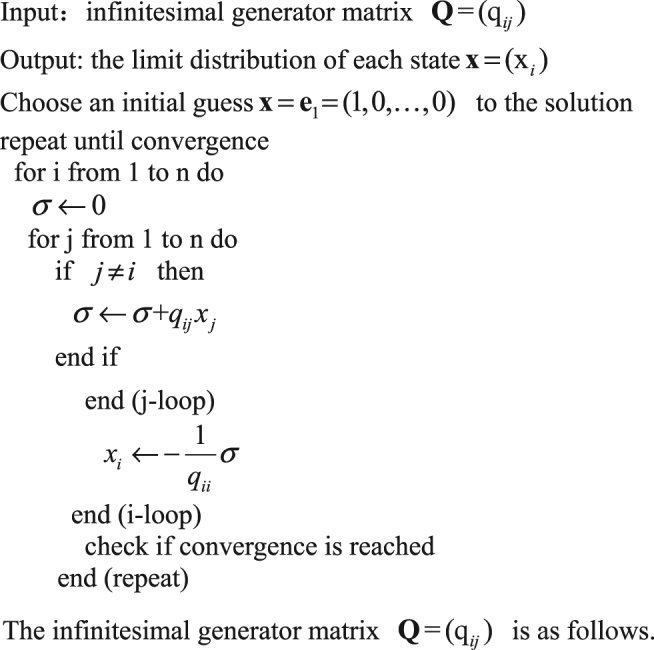
The Gauss-Seidel iterative algorithm.

The infinitesimal generator matrix ${\bf{Q}}={(q}_{ij})$ is as follows.

$${\bf{Q}}=(\begin{array}{ccccc}{A}_{0} & {C}_{0} &  &  & \\ {B}_{1} & {A}_{1} & {C}_{1} &  & \\  & \ddots  & \ddots  & \ddots  & \\  &  & {B}_{M-1} & {A}_{M-1} & {C}_{M-1}\\  &  &  & {B}_{M} & {A}_{M}\end{array})$$

where (the blank of each matrix equals zero)

$${{\rm{A}}}_{i}=(\begin{array}{ccccc}{D}_{i,0} & {F}_{i,0} &  &  & \\ {E}_{i,1} & {D}_{i,1} & {F}_{i,1} &  & \\  & \ddots  & \ddots  & \ddots  & \\  &  & {E}_{i,M-i-1} & {D}_{i,M-i-1} & {F}_{i,M-i-1}\\  &  &  & {E}_{i,M-\iota } & {D}_{i,M-i}\end{array})$$

$${D}_{i,j}=(\begin{array}{ccccc}{d}_{i,{\rm{j}},0} & {p}_{L\to P}^{({\rm{i}},{\rm{j}},0)} &  &  & \\ {p}_{P\to L}^{({\rm{i}},{\rm{j}},1)} & {d}_{i,{\rm{j}},1} & {p}_{L\to P}^{({\rm{i}},{\rm{j}},1)} &  & \\  & \ddots  & \ddots  & \ddots  & \\  &  & {p}_{P\to L}^{({\rm{i}},{\rm{j}},{\rm{M}}-{\rm{i}}-{\rm{j}}-1)} & {d}_{i,{\rm{j}},{\rm{M}}-{\rm{i}}-{\rm{j}}-1} & {p}_{L\to P}^{({\rm{i}},{\rm{j}},{\rm{M}}-{\rm{i}}-{\rm{j}}-1)}\\  &  &  & {p}_{P\to L}^{({\rm{i}},{\rm{j}},{\rm{M}}-{\rm{i}}-{\rm{j}})} & {d}_{i,{\rm{j}},{\rm{M}}-{\rm{i}}-{\rm{j}}}\end{array})$$

$${F}_{i,j}=(\begin{array}{cccc}{p}_{L\to D}^{({\rm{i}},{\rm{j}},0)} &  &  & \\ {p}_{P\to D}^{({\rm{i}},{\rm{j}},{\rm{1}})} & {p}_{L\to D}^{({\rm{i}},{\rm{j}},{\rm{1}})} &  & \\  & {p}_{P\to D}^{({\rm{i}},{\rm{j}},2)} & \ddots  & \\  &  & \ddots  & {p}_{L\to D}^{({\rm{i}},{\rm{j}},{\rm{M}}-{\rm{i}}-{\rm{j}}-1)}\\  &  &  & {p}_{P\to D}^{({\rm{i}},{\rm{j}},{\rm{M}}-{\rm{i}}-{\rm{j}})}\end{array})$$

$${E}_{i,j}=(\begin{array}{ccccc}{p}_{D\to L}^{({\rm{i}},{\rm{j}},0)} & {p}_{D\to P}^{({\rm{i}},{\rm{j}},0)} &  &  & \\  & {p}_{D\to L}^{({\rm{i}},{\rm{j}},{\rm{1}})} & {p}_{D\to P}^{({\rm{i}},{\rm{j}},{\rm{1}})} &  & \\  &  & \ddots  & \ddots  & \\  &  &  & {p}_{D\to L}^{({\rm{i}},{\rm{j}},{\rm{M}}-{\rm{i}}-{\rm{j}})} & {p}_{D\to P}^{({\rm{i}},{\rm{j}},{\rm{M}}-{\rm{i}}-{\rm{j}})}\end{array})$$

$${B}_{i}=(\begin{array}{ccccc}{G}_{i,0} & {H}_{i,0} &  &  & \\  & {G}_{i,1} & {H}_{i,1} &  & \\  &  & \ddots  & \ddots  & \\  &  &  & {G}_{i,{\rm{M}}-{\rm{i}}} & {H}_{i,{\rm{M}}-{\rm{i}}}\end{array})$$

$${G}_{i,j}=(\begin{array}{ccccc}{p}_{C\to L}^{({\rm{i}},{\rm{j}},0)} & {p}_{C\to P}^{({\rm{i}},{\rm{j}},0)} &  &  & \\  & {p}_{C\to L}^{({\rm{i}},{\rm{j}},{\rm{1}})} & {p}_{C\to P}^{({\rm{i}},{\rm{j}},{\rm{1}})} &  & \\  &  & \ddots  & \ddots  & \\  &  &  & {p}_{C\to L}^{({\rm{i}},{\rm{j}},{\rm{M}}-{\rm{i}}-{\rm{j}})} & {p}_{C\to P}^{({\rm{i}},{\rm{j}},{\rm{M}}-{\rm{i}}-{\rm{j}})}\end{array})$$

$${H}_{i,j}=(\begin{array}{cccc}{p}_{C\to D}^{({\rm{i}},{\rm{j}},0)} &  &  & \\  & {p}_{C\to D}^{({\rm{i}},{\rm{j}},{\rm{1}})} &  & \\  &  & \ddots  & \\  &  &  & {p}_{C\to D}^{({\rm{i}},{\rm{j}},{\rm{M}}-i-j)}\end{array})$$

$${C}_{i}=(\begin{array}{cccc}{K}_{i,0} &  &  & \\ {I}_{i,1} & {K}_{i,1} &  & \\  & {I}_{i,2} & \ddots  & \\  &  & \ddots  & {K}_{i,{\rm{M}}-i-1}\\  &  &  & {I}_{i,{\rm{M}}-i}\end{array})$$

$${K}_{i,j}=(\begin{array}{cccc}{p}_{L\to C}^{({\rm{i}},{\rm{j}},0)} &  &  & \\ {p}_{P\to C}^{({\rm{i}},{\rm{j}},{\rm{1}})} & {p}_{L\to C}^{({\rm{i}},{\rm{j}},{\rm{1}})} &  & \\  & {p}_{P\to C}^{({\rm{i}},{\rm{j}},2)} & \ddots  & \\  &  & \ddots  & {p}_{L\to C}^{({\rm{i}},{\rm{j}},{\rm{M}}-{\rm{i}}-{\rm{j}}-{\rm{1}})}\\  &  &  & {p}_{P\to C}^{({\rm{i}},{\rm{j}},{\rm{M}}-{\rm{i}}-{\rm{j}})}\end{array})$$

$${I}_{i,j}=(\begin{array}{cccc}{p}_{D\to C}^{({\rm{i}},{\rm{j}},0)} &  &  & \\  & {p}_{D\to C}^{({\rm{i}},{\rm{j}},{\rm{1}})} &  & \\  &  & \ddots  & \\  &  &  & {p}_{D\to C}^{({\rm{i}},{\rm{j}},{\rm{M}}-i-j)}\end{array})$$

The ${d}_{i,j,k}(i,j,k=0,1,\ldots ,M\,and\,i+j+k\le M)$ of ${\bf{Q}}={(q}_{ij})$ can be obtained by equations ${\bf{Q}}1={\bf{0}}$, where **1** is the column vector with all components equal to one, and **0** is the column vector with all components equal to zero.

## Electronic supplementary material

Supplementary Information
